# Maxillofacial fracture epidemiology and treatment plans in the Northeast of Iran: A retrospective study

**DOI:** 10.4317/medoral.21809

**Published:** 2017-08-16

**Authors:** Sahand Samieirad, Mohammad-Reza Aboutorabzade, Elahe Tohidi, Baratollah Shaban, Hussein Khalife, Maryam-Alsadat Hashemipour, Hamid-Reza Salami

**Affiliations:** 1 Assistant Professor, Oral & Maxillofacial Diseases Research Center, Mashhad University of Medical Sciences, Mashhad, Iran; 2 Student Research Committee, Faculty of Dentistry, Mashhad University of Medical Sciences, Mashhad, Iran; 3 Assistant Professor, Oral & Maxillofacial Diseases Research Center, Mashhad University of Medical Sciences, Mashhad, Iran; 4 DMD, MD, Private Practice Beirut, Lebanon; 5 DDS, MSc, Member of Kerman Dental and Oral Diseases Research Center, Associate Professor, Department of Oral Medicine, Dental School, Kerman University of Medical Science, Kerman, Iran

## Abstract

**Background:**

The epidemiology of facial injuries varies based on lifestyle, cultural background and socioeconomic status in different countries and geographic zones. This study evaluated the epidemiology of maxillofacial fractures and treatment plans in hospitalized patients in Northeast of Iran (2015-2016).

**Material and Methods:**

In this retrospective study, the medical records of 502 hospitalized patients were evaluated in the Department of Maxillofacial Surgery in Kamyab Hospital in Mashhad, Iran. The type and cause of fractures and treatment plans were recorded in a checklist. Data were analyzed with Mann–Whitney test, chi-squared test and Fisher’s exact test, using SPSS 21.

**Results:**

The majority of patients were male (80.3%). Most subjects were in 20-30-year age range (43.2%). The fractures were mostly caused by accidents, particularly motorcycle accidents (MCAs), and the most common site of involvement was the body of the mandible. There was a significant association between the type of treatment and age. In fact, the age range of 16-59 years underwent open reduction internal fixation (ORIF) more than other age ranges (*P*=0.001). Also, there was a significant association between gender and fractures (*P*=0.002).

**Conclusions:**

It was concluded that patient age and gender and trauma significantly affected the prevalence of maxillofacial traumas, fracture types and treatment plans. This information would be useful for making better health policy strategies.

** Key words:**Epidemiology, treatment, facial injuries, maxillofacial fractures, trauma.

## Introduction

An increase in population in cities and industrial development has resulted in changes in lifestyles and personal activities. These changes result in increasing rate of injuries, especially maxillofacial fractures (Fx) owing to the specific anatomical features of this region (1,2). These injuries are one of the most common issues dealt with by both maxillofacial and plastic surgeons in their professional practice (3).

These fractures might give rise to socioeconomic burden and deleterious effects on both the community and health system. These injuries are among the major health concerns worldwide (4,5). Furthermore, treatment and rehabilitation of maxillofacial fractures are associated with psychological and esthetic concerns, severe morbidity and disabilities. In addition, these traumas would impose a significant financial burden on individuals and societies (6,7). Therefore it is necessary to pay more attention to their epidemiology and details.

In most parts of the world, major causes of facial fractures are motor vehicle accidents (MVA), falls, assaults, sports and occupational injuries (5,8). Epidemiology and pattern of soft and hard facial tissue injuries vary in different societies as a function of cultural and socioeconomic factors (9).

Several studies have investigated the epidemiology of maxillofacial fractures in different countries and populations (10-15). However, there is still limited data regarding the epidemiology and treatment plans of facial injuries in developing countries, especially in Iran.

Some researchers have investigated the prevalence of maxillofacial fractures in different provinces and regions of Iran (16-18); for example, the senior author has already studied the maxillofacial fracture epidemiology in the southeast of Iran (5). However, there is still a lack of sufficient information about the etiology, prevalence, epidemiology and outcomes of these injuries, especially in the northeast of Iran as a result of its specific socio-political and religious conditions.

According to the literature, MVAs are the most common cause of maxillofacial fractures in Iran, like other developing countries (5,19-22). However, assaults are the dominant casual factors for MVAs in developed countries (5,23). This difference is attributed to differences in safety driving rules (24).

Mashhad as the Capital of Khorasan Province receives the maximum number of passengers and pilgrims annually and due to its short distance from Afghanistan, a lot of road accidents, assaults and gunshots take place there (25). These victims are mainly transferred or referred to Trauma Emergency Center of Shahid Kamyab Hospital since this hospital is a major educational and therapeutic multiple trauma center in Khorasan.

Epidemiologic investigations and study of the factors in the region are very important. The present study in the second one which is done in Iran and according to numerous accidents in developing countries, especially Iran, pay attention to the factor of fractures by accidents is very essential. Therefore, the present study was undertaken to develop and analyze the available epidemiological and statistical data related to facial fractures in Iran and also to evaluate the incidence of maxillofacial fractures in hospitalized patients in terms of age, gender, types and causes of trauma and treatment plans in the Oral and Maxillofacial Surgery Department of Shahid Kamyab Hospital in Mashhad, located in the northeast of Iran during 2015-2016 by considering clinical, demoghraphic and radiographic data.

## Material and Methods

The study was designed as a retrospective cross-sectional study with the ethical code IR.mums.sd.REC.1394.127. The study was approved by the Institutional Human Research and Ethics Committee of Mashhad University of Medical Sciences, Mashhad, Iran.

This was a census-based study to assess the prevalence, types and causes of trauma in patients with maxillofacial fractures. Therefore, all the patients with a diagnosis of maxillofacial fracture, who were admitted and treated in Shahid Kamyab Hospital, Mashhad, Iran in 2015-2116, were included in the study. The sample size was calculated at 561 cases according to the admission office information. Ethical considerations were taken into account throughout the study, and the patients’ names and medical information remained completely confidential.

The exclusion criteria were as follows: 1) incomplete medical records; 2) patients with only dentoalveolar fractures undergoing reduction by arch bar without hospitalization; 3) patients with only soft tissue injuries, who were treated in the emergency room without hospitalization; 4) patients undergoing other procedures such as opening the arch bar, or removal of a plate in patients who underwent maxillofacial surgeries before. After excluding these cases, only 502 patients remained to be analyzed. All the demographic data (e.g. patients’ age and gender) were collected, and the patients’ medical records were examined to extract information related to the date of referral, cause of trauma, the affected bones, concomitant fractures and injuries of other organs, the exact maxillo-mandibular status, facial examinations, and radiographic images. Data collection tools included observation and census sampling of medical records and documents and also PACS (picture archiving and communicating system) and archived radiology reports data in the surgery ward of the hospital. The patients’ methods of treatment were evaluated and surveyed in this study. Maxillofacial fractures were treated using the following methods in our department: 1) closed reduction (CR); 2) open surgical treatment or open reduction and internal fixation (ORIF); 3) combination therapy (both CR and ORIF), 4) follow-up and re-evaluation of the status of suspected fractures (without any specific treatments).

Then this data was imported to SPSS 21. We used descriptive statistics such as distribution and continuity (means and standard deviations) for representing the data collected.

For statistical analysis the significance level was set at 0.05; Mann–Whitney test was performed to compare differences in consequences among females and males and chi-squared test and Fisher’s exact test were used to analyze the relations between qualitative parameters.

## Results

A total of 502 patients were evaluated in this study; 403 subjects (80.3%) were male and 99 (19.7%) were female. The mean age of the subjects was 28.8±13.56 years (age range: 2-80 years). The majority of cases were within the age range of 20-30 (43.2%) years, followed by 30-40 (16.7%) years and 10-20 (16.1%), respectively ( Table 1).

**Table 1:**
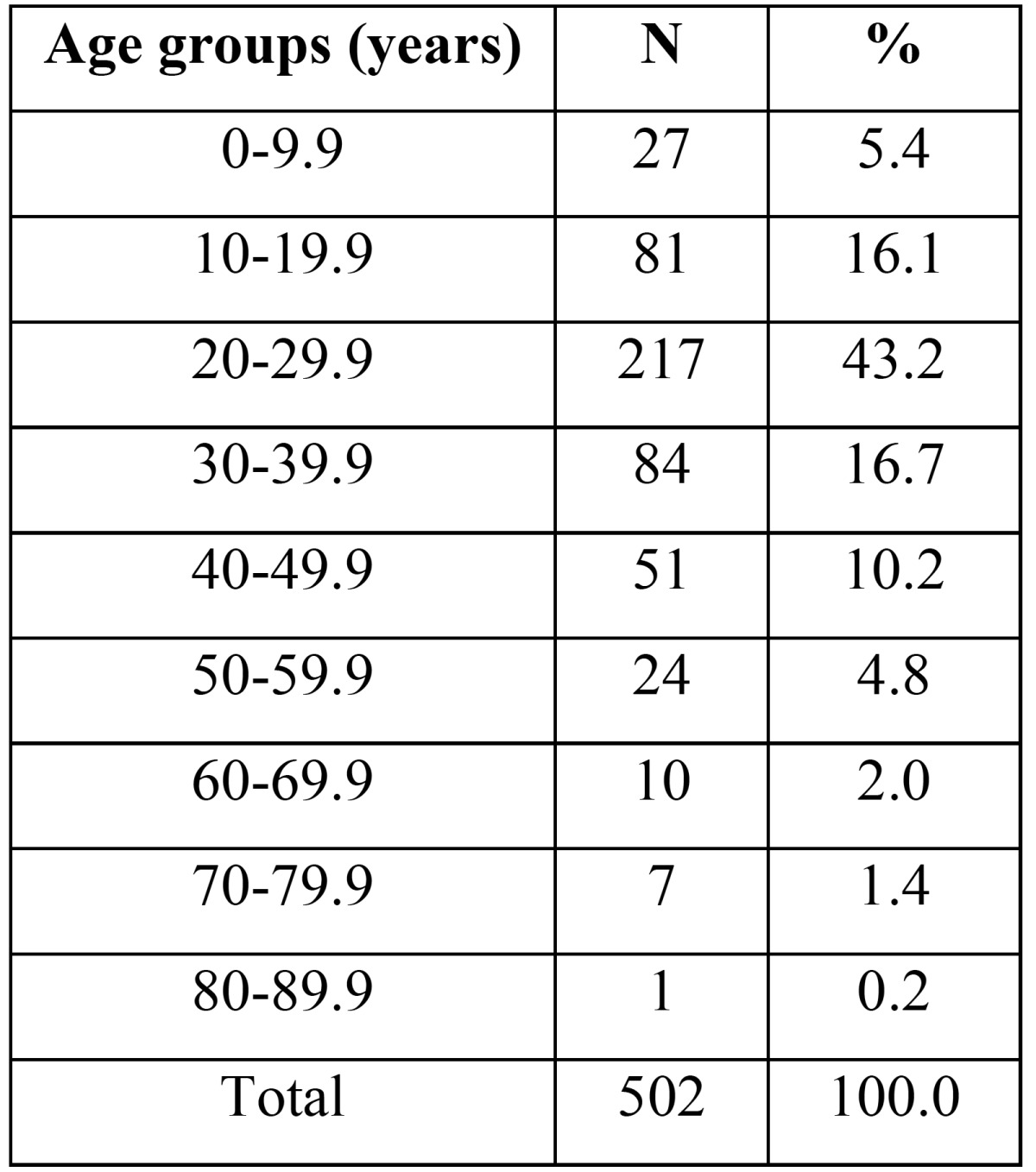
Frequency of maxillofacial fractures in different age ranges.

The highest rate of fractures occurred in summer (29.1%). In fact, the highest rates were reported in September (11.9%) and October (10.2%), followed by April (10.0%). In total, 278 and 224 cases underwent treatment in 2015 and 2016, respectively. MCAs (motor cycle accidents) accounted for the majority of traumas (62.7%) and CAs (car accidents) (17.9%) ranked the second. A total of 832 anatomic and bone fractures were found in 502 patients, and in total, 1049 cases of fracture lines were reported.

In relation to categorization of fractures, 173 cases (34.5%) had simple fractures and 329 subjects (65.5%) had multiple fractures, while 39.4 % of cases had two lines of fracture.

As a matter of fact, the total percentage of fractures in anatomic locations was higher than 100%, given the possibility of having fractures in several locations.

The authors also determined the anatomical location of maxillofacial fractures. Among 502 patients evaluated, mandibular fractures had the highest frequency (58.8%), followed by zygomatico-maxillary complex (ZMC) fractures (36.7%) and the nasal bone (18.33%) ( Table 2).

**Table 2:**
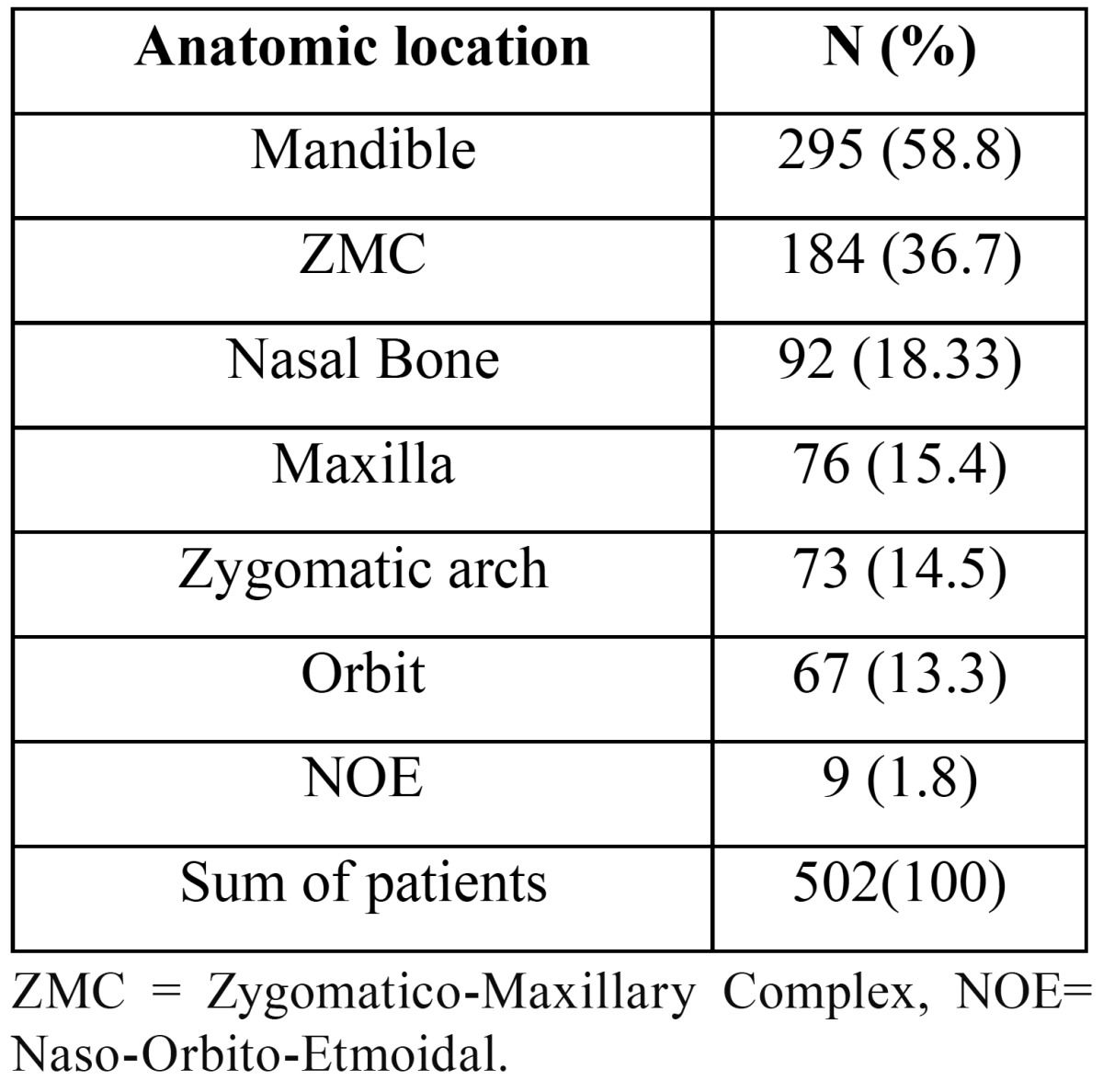
Frequency and percentage of the anatomical location of maxillofacial fractures in 502 patients.

It should be noted that among 502 cases with 832 anatomic bone fractures, there were 295 patients with 426 different mandibular fractures. The most common anatomical location of fracture in the mandible was the body (39.67%), followed by parasymphysis (20.19%) and subcondylar fractures (16.67%), respectively. The lowest number of fractures was recorded in the coronoid area (1.17%) ( Table 3).

**Table 3:**
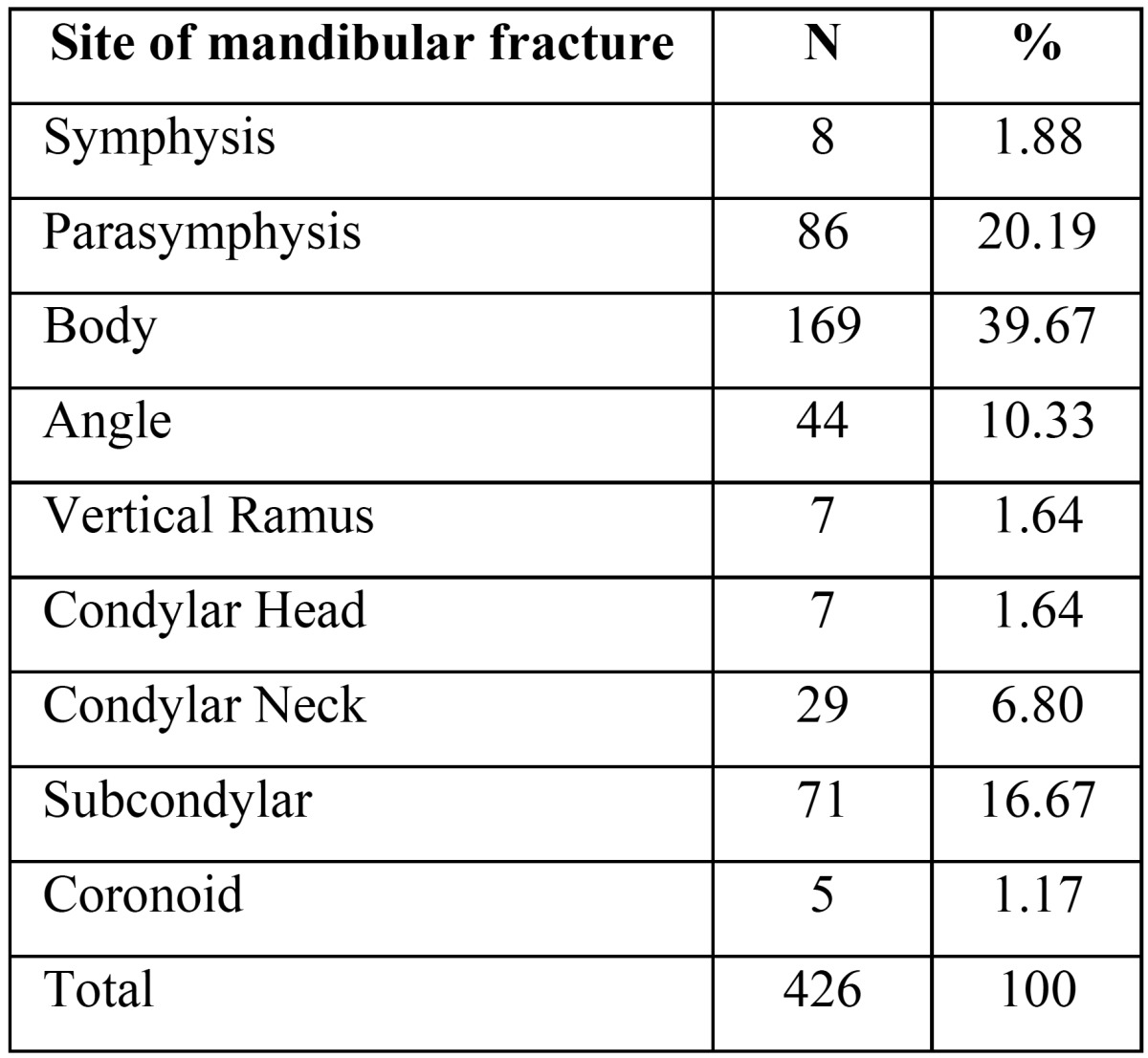
The frequency of the anatomical locations of mandibular fractures.

Based on Peterson’s classification (20), if parasymphysis is considered as a portion of symphysis, fracture frequency would be estimated at 22.7%. If the head and neck of condyle and subcondylar region are considered as a single component, the overall incidence of condylar fractures would be 25.12%.

**Figure 1:**
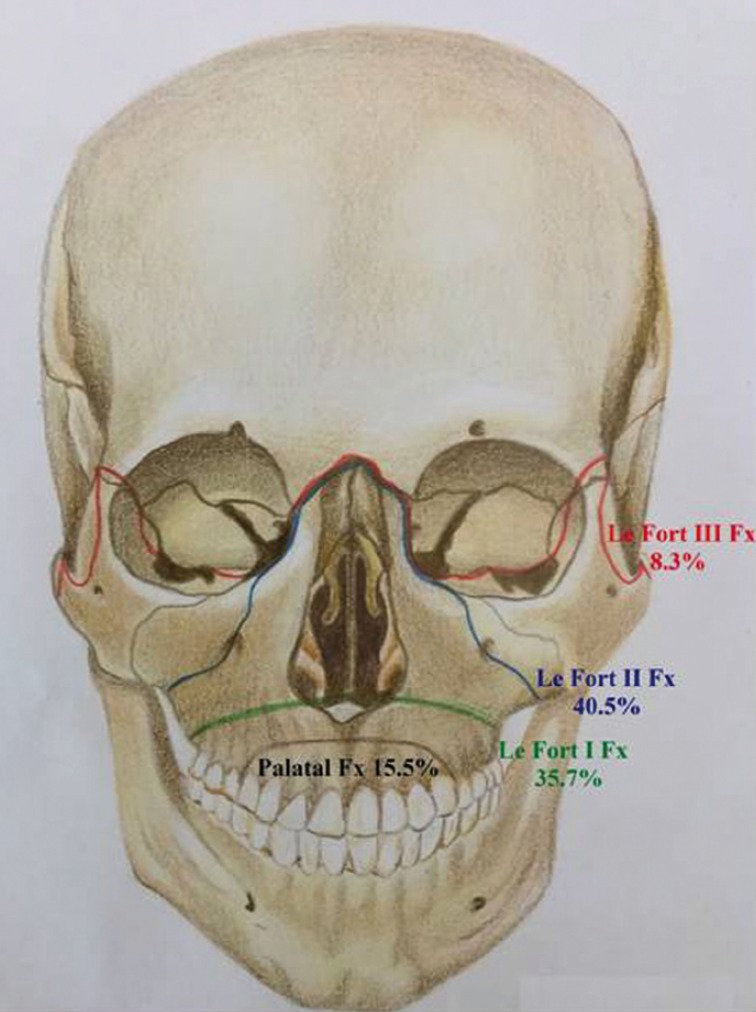
The frequency and classification of maxillary fractures.

A total of 84 fracture lines were observed in 76 patients with maxillary fractures. The most commonly reported site in patients with fractured maxilla was LeFort II (the maxilla separates from the face) with a prevalence of 40.48%, followed by LeFort I (the palate is separated from the maxilla) and LeFort III (craniofacial disjunction is present), with frequencies of 35.71% and 8.33%, respectively (Fig. 1).

Simultaneous injuries were reported in 158 patients (31.47%). The most common concomitant injuries were orthopedic fractures, reported in 71.5% of the patients, followed by cranial fractures with 29.47%.

Furthermore, in these cases, the most frequent treatment was ORIF (43%), followed by a combination of CR and ORIF (33%) and CR (24%), respectively. It was possible to perform both CR and ORIF for several fractures in one patient simultaneously; in other word, the treatment plans would be 57.5% ORIF and 42.5% CR of all the treatments.

Table 4 shows the frequencies of maxillofacial treatment plans in terms of fracture sites ( Table 4).

**Table 4:**
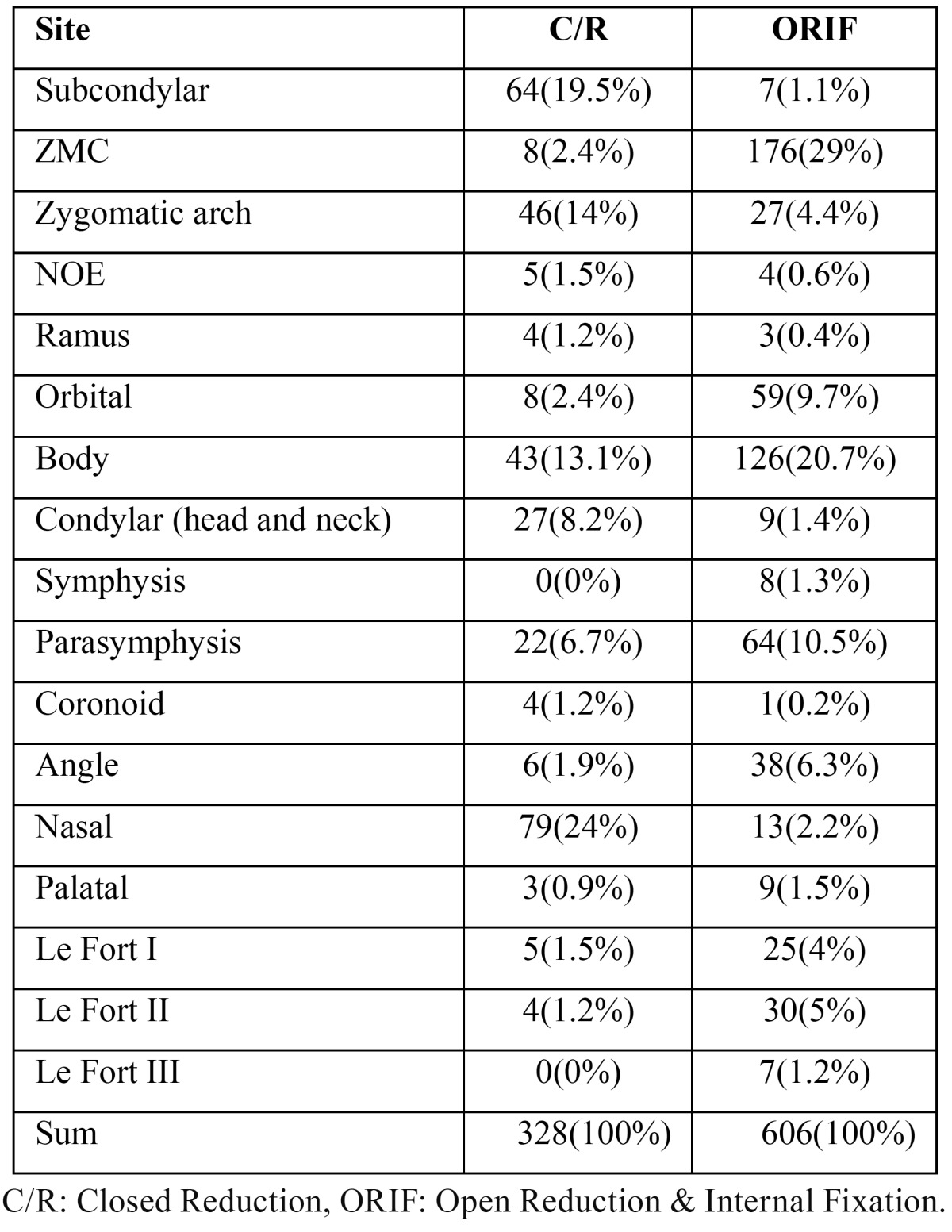
Frequency and percentage of maxillofacial treatment plans regarding to fractures sites.

According to the results, the ORIF treatments were performed more commonly for ZMC (29%) and mandibular body fractures (20.8%). However, the closed approach was a more prevalent treatment plan for nasal bone (24.1%) and subcondylar fractures (19.5%) (*P*=0.001).

Table 5 shows the association between maxillofacial fractures and gender and type of fracture ( Table 5).

**Table 5:**
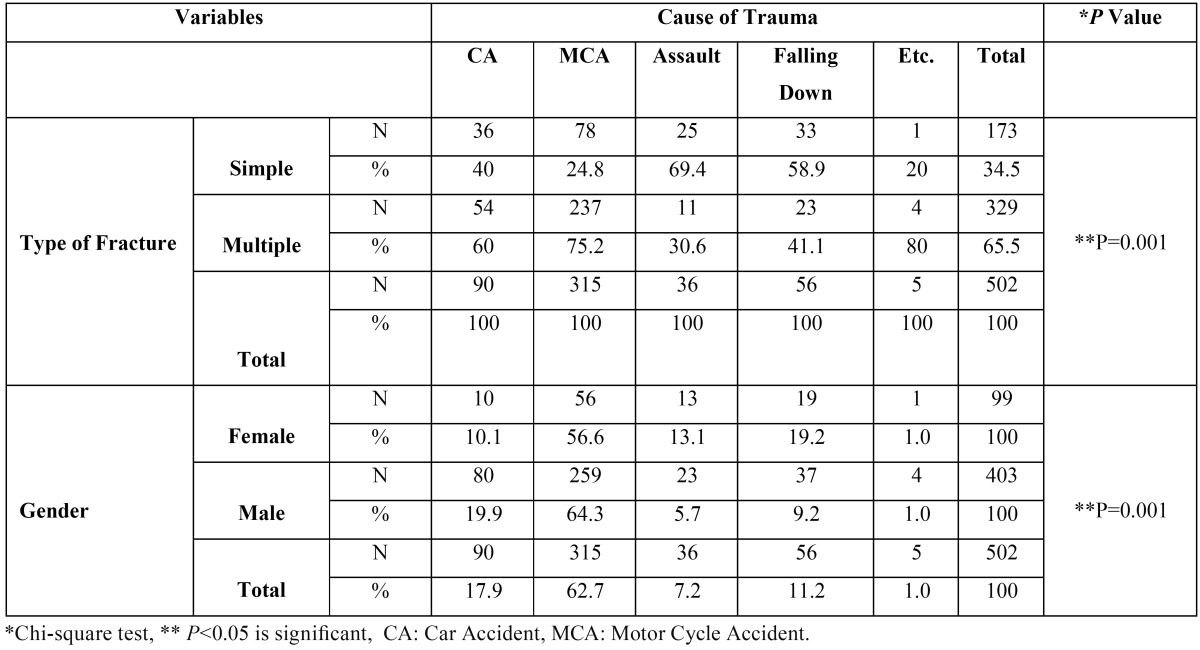
Association between the cause of maxillofacial fractures, gender, and type of fracture.

The prevalence of fractures in males was 4.07 times higher than females. There was no significant age difference between male (28.98±15.65) and female patients (28.10±13.01) according to Mann-Whitney test (*P*=0.423, Z=0.80).

A significant association was observed between gender and the cause of fractures (*P*=0.001). Males were more prone to MVAs and assaults, compared to females. In cases of assaults and falls, the fracture types were simple and isolated, while in car accidents and especially motorcycle accidents, most fractures were multiple. In this regard, chi-squared test showed a significant association between the type of fractures and cause of trauma (*P*=0.001) ( Table 5).

The findings showed that most maxillofacial treatment plan were open reduction (57.5%), followed by closed reduction (42.5%) in our department. In addition, in the age category of <15 years, most maxillofacial treatment plans were CR (58%); CR was also reported in the age category of >60 years (81%). However, in the age range of 16-59 years, ORIF was the predominant treatment method (60.5%). Chi-squared test showed a significant difference between the type of treatment and age; in fact, the age range of 16-59 years underwent open treatment more than other age ranges (*P*=0.002) ( Table 6).

**Table 6:**
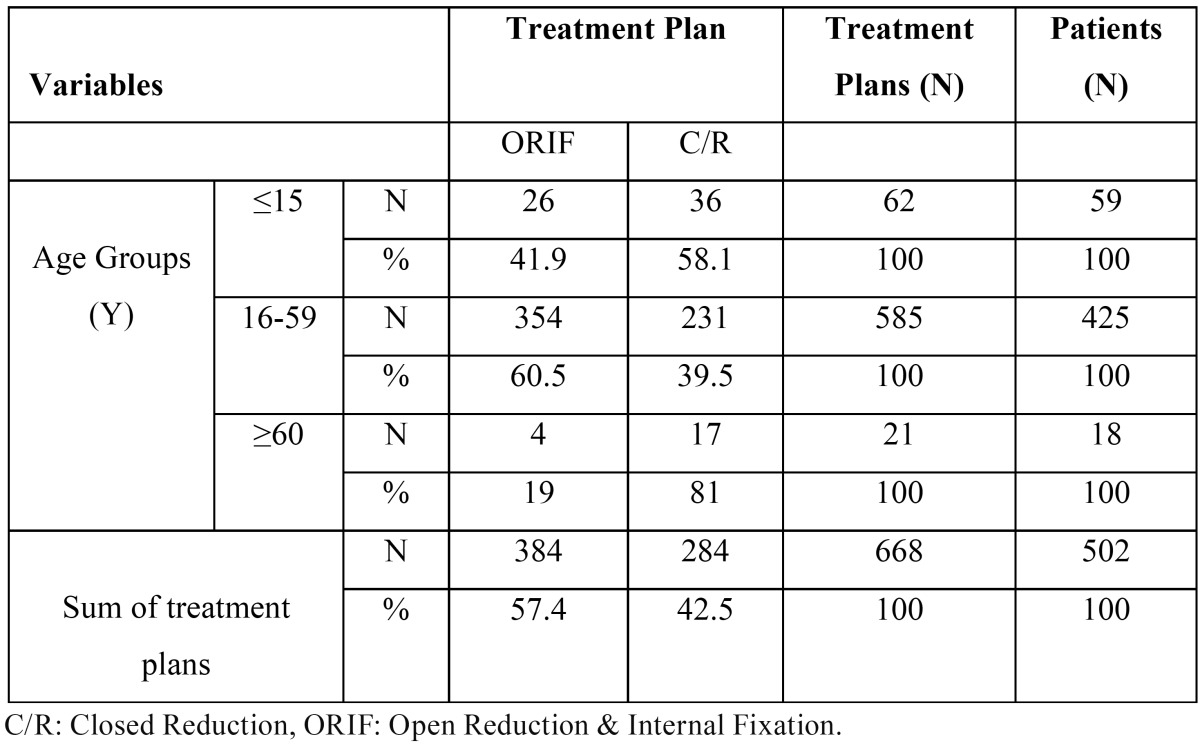
Frequency of treatment plans according to patients’ age categories.

## Discussion

In addition to the possible life-threatening nature of facial traumas, these injuries might cause esthetic or functional deformities which might lead to psychological, financial and social costs for people and society (5). Therefore, it is of high significance to identify the etiology and epidemiology and treatment plans of maxillofacial traumas.

Mashhad, the capital of Khorasan Razavi Province, is the second largest and most developed city in Iran with a population of approximately 6 million in the last census (2011) (26,27). This city is important because of its religious nature and hosts a large number of passengers and Muslim pilgrims from other cities and also neighboring countries each year (25).

The Oral and Maxillofacial Surgery Department of Kamyab Hospital of Mashhad is the most equipped and highly advanced maxillofacial surgery department in the northeast of Iran.

Most of the epidemiological studies on maxillofacial fractures have been performed retrospectively (3-6,11,16,19,20,28-35).

During 2 years, from January 2015 to December 2016, 502 patients were treated by the oral and maxillofacial surgery staff and residents in our department. In our study, the patient-to-year ratio was higher than what has been reported in most investigations into the prevalence of maxillofacial fractures in Iran (5,16,18-20,22,28,36), Italy (37), India (32,33,38), Brazil (4,39,40), Netherlands (41) and Serbia (42).

The analysis of this sample might provide useful knowledge about the current distribution of facial fractures in the northeast of Iran, as well as offering a new valuable health care system database that might improve medical and dental policies to prevent and manage facial trauma.

As we reported, the incidence rate of fractures in men was higher than that in women (4.07/1). This finding was consistent with several previously conducted studies by Lee, Cabalag, Balli and Paes *et al.* (4,38,43,44), as males are generally more socially active and more involved in life-threatening activities, careless fast car and motorcycle driving, sports and violence.

The highest number of injuries was reported in the age range of 20-30 years, consistent with other studies (4,5,10,12,16,18,21,23,35,38).

Considering the careless and fast driving attitude without the use of safety belts or helmets in this age range and also the interest in adventures and assaults among the youth, they are more prone to traumas.

Based on the results, the highest number of traumas was noted in September, October and April, consistent with the findings by other studies (5,12,28,41,51-53).

This result can be explained by the fact that Iranian people tend to be more exposed to trauma risk factors in the summer and spring, because of engaging more frequently in physical activities, taking part in social Norouz reunions and summer holiday road trips. In addition, motorcycle accidents were common in the summer, which can be due to difficulty of helmet wearing in hot weather (54).

According to Huelke and Compton, although car accidents are more frequent, motorbike accidents are usually more severe (55).

Our study showed that MCAs were the most common cause of fractures (62.7%), followed by CA (17.9%). This would be explained by high-speed and careless driving in Mashhad, along with disrespect for traffic laws and the frequent disregard for wearing helmets.

This finding was consistent with the results of most Iranian studies (5,21,36) and some studies in other countries (10,30,31,50,51,55).

However, cultural differences, sports activities, daily tasks, occupational status and strict driving rules might affect the etiology of maxillofacial traumas, leading to discrepancies between various studies.

Our findings showed that the mandible was the most involved bone (58.8%), consistent with other studies (5,10,12,17,31,32,39,50).

Some other researchers such as Mohajerani, Arangio, Van Hout and VanAs et al. found that facial fractures in the zygomatic complex were more frequent (21,37,41,56). ZMC fracture was the second most frequent fracture in our study.

Zandi, Momeni and Hussain *et al.* showed that nasal bone fractures were the most prevalent type of trauma (22,36,57), which were the third most prevalent maxillofacial fracture in the present study.

Minor differences in the frequency of fractures can be caused by variations in the etiology of fractures in various regions and the difference in patients’ referral system.

As the MVAs with their high-energy impact were the most frequent cause of trauma in our research, multiple fractures were more prevalent (65.5%) than isolated ones. This finding is consistent with the results of a study by Samieirad *et al.* in Kerman (5).

Lee and Anbiaee *et al.* showed that mandibular body region accounted for the highest number of fractures (25,44), consistent with our results. However, Balli and Samieirad *et al.* reported parasymphysis as the most prevalent site (5,38), which ranked the second in our research.

Our findings are compatible with those of a study by Motamedi *et al.* as they reported that Lefort II fractures were most frequent in the maxilla (18).

Momeni and Samieirad *et al.* reported orthopedic injuries as the most common associated trauma (5,36), consistent with the current findings indicating that orthopedic injuries (71.5%) were the most prevalent concomitant injuries.

The most prevalent method of treatment in our department was open reduction and internal fixation (ORIF) (57.5%), consistent with other results (38,40,43,44).

No complications concerning occlusion and mouth opening and even sensory nerve paresthesia and infection were encountered in these patients.

Recently, plate osteosynthesis has become popular in the management of facial fractures and in the treatment of mandibular fractures all over the world, especially in Iran (5,38). Surgeons prefer ORIF because it offers the advantages of stable and precise anatomical reduction of fragments, allows immediate recovery of function as it has no IMF (inter-maxillary fixation). This treatment plan would decrease the period of bone healing and the recovery period (5).

The treatment plan selection is based on patients’ age and also anatomic location of fractures. In our department, ORIF surgeries are performed routinely for ZMC and mandibular body fractures; however, most of the nasal and mandibular subcondylar fractures are treated with closed approach.

This treatment plan is consistent with scientific protocols for the management of facial fractures in order to achieve the best functional and esthetic outcomes with the least scars and sensory or motor nerve complications (58-62).

It is of interest that patients under 15 and over 60 years of age were treated mostly by closed reduction in this study. These findings were compatible with the results of studies by Samieirad, Zandi and Kambalimath *et al.* (5,22,33).

Because of high osteogenic potential in pediatric age, even ORIF might increase the risk of tooth bud injuries and also induce developmental asymmetry. Therefore, closed reduction can be a good therapeutic choice (33,63). Considering the low repairing capacity and systemic health problems in geriatric ages, ORIF treatment has its own complications (64,65). Therefore, patients in extreme age ranges would benefit from closed reduction treatment plans.

The following simplified message are made after analysis of the results of the present study.

●Accidents are a serious public health problem in Iran because of a variety of reasons including young population of the country which leads to more exposure to accident, low gas price, decreased ratio of transports by public transportation than with private vehicles, and non-standard safety designs (66).

●Accidents and its related injuries contribute to a significant proportion of the burden of diseases in Iran. They also have a significant impact on the social and economic well-being of people. Also, the worldwide road traffic injury/death rate is 3 people per 10 000 vehicles, but in Iran it is 33 people per10 000 vehicles (67).

●Epidemiologic investigations and study of the factors in the region are very important. The present study in the second one which is done in Iran and according to numerous accidents in developing countries, especially Iran, pay attention to the factor of fractures by accidents is very essential.

●In the present study an attempt is made to investigate all of factors lead to fractures in accidents and same curing treatment is studied.

●Mashhad, the capital of Khorasan Razavi Province, is the second largest and most developed city in Iran with a population of approximately 6 million in the last census. This city is important because of its religious nature and hosts a large number of passengers and Muslim pilgrims from other cities and also neighboring countries each year.

## Conclusions

It can be concluded that patients’ age, gender and also trauma causes would significantly affect the prevalence of maxillofacial traumas, fracture types and also the decision about the best treatment plan. This would be beneficial for appropriate health care policy and management set-up in every developing society for education, prevention and treatment.

## References

[1] Andreas ZJ, Benoit S, Olivier L, Nikola S, Hanna T, Tateyuki I (2011). Incidence, aetiology and pattern of mandibular fractures in central Switzerland. Swiss Med Wkly.

[2] Dongas P, Hall G (2002). Mandibular fracture patterns in Tasmania, Australia. Aus Dental J.

[3] Ferreira PC, Amarante JM, Silva PN, Rodrigues JM, Choupina MP, Silva ÁC (2005). Retrospective study of 1251 maxillofacial fractures in children and adolescents. PlaRecon Surg.

[4] Paes JV, de Sa Paes FL, Valiati R, de Oliveira MG, Pagnoncelli RM (2012). Retrospective study of prevalence of face fractures in southern Brazil. Indian J Dent Res.

[5] Samieirad S, Tohidi E, Shahidi-Payam A, Hashemipour M A, Abedini A (2015). Retrospective study maxillofacial fractures epidemiology and treatment plans in Southeast of Iran. Med Oral Patol Oral Cir Bucal.

[6] Aksoy E, Ünlü E, Sensöz Ö (2002). A retrospective study on epidemiology and treatment of maxillofacial fractures. J CranioSurg.

[7] Wittchen HU, Jacobi F, Rehm J, Gustavsson A, Svensson M, Jönsson B (2011). The size and burden of mental disorders and other disorders of the brain in Europe 2010. Eur Neuropsych Pharmacol.

[8] Wood E, Freer T (2001). Incidence and Aetiology of Facial Injuries Resulting from Motor Vehicle Accidents in Queensland for a Three‐year Period. Aus Dental J.

[9] Ribeiro M, Marcenes W, Croucher R, Sheiham A (2004). The prevalence and causes of maxillofacial fractures in patients attending Accident and Emergency Departments in Recife‐Brazil. Inter DentJ.

[10] Adebayo ET, Ajike O, Adekeye E (2003). Analysis of the pattern of maxillofacial fractures in Kaduna, Nigeria. Br J Oral Maxillo Surg.

[11] Al-Khateeb T, Abdullah FM (2007). Craniomaxillofacial injuries in the United Arab Emirates: a retrospective study. J Oral Maxillo Surg.

[12] Brasileiro BF, Passeri LA (2006). Epidemiological analysis of maxillofacial fractures in Brazil: a 5-year prospective study. Oral Surg Oral Med Oral Pathol Oral Radiol Endod.

[13] Erol B, Tanrikulu R, Görgün B (2004). Maxillofacial Fractures. Analysis of demographic distribution and treatment in 2901patients (25-year experience). J Cranio Surg.

[14] Fasola AO, Nyako EA, Obiechina AE, Arotiba JT (2003). Trends in the characteristics of maxillofacial fractures in Nigeria. J Oral Maxillo Surg.

[15] Moncrieff NJ, Qureshi C, Deva AK (2004). A comparative cost analysis of maxillofacial trauma in Australia. J Cranio Surg.

[16] Ansari MH (2004). Maxillofacial fractures in Hamedan province, Iran: a retrospective study (1987–2001). J Cranio Surg.

[17] Kadkhodaie M (2006). Three-year review of facial fractures at a teaching hospital in northern Iran. BrJ Oral Maxillo Surg.

[18] Motamedi MHK (2003). An assessment of maxillofacial fractures: a 5-year study of 237 patients. J Oral Maxillo Surg.

[19] Hashemi HM, Beshkar M (2011). The prevalence of maxillofacial fractures due to domestic violence–a retrospective study in a hospital in Tehran, Iran. Dent Traumatol.

[20] Mesgarzadeh AH, Shahamfar M, feizi Azar S, Shahamfar J (2011). Analysis of the pattern of maxillofacial fractures in north western of Iran: A retrospective study. J Emer Trauma Shock.

[21] Mohajerani SH, Asghari S (2011). Pattern of mid‐facial fractures in Tehran, Iran. Dent Traumatol.

[22] Zandi M, Khayati A, Lamei A, Zarei H (2011). Maxillofacial injuries in western Iran: a prospective study. Oral Maxillo Surg.

[23] Adeyemo WL, Ladeinde AL, Ogunlewe MO, James O (2005). Trends and characteristics of oral and maxillofacial injuries in Nigeria: a review of the literature. Head Face Med.

[24] Banakar R, Fard SN (2012). Driving dangerously: law, culture and driving habits in Iran. Br J Mid Eastern Stud.

[25] Anbiaee N, Vaezi T, Khamchin F, Hafez Maleki F (2015). Maxillofacial Fractures in CT scan Images of Adult, Adolescent, and Child Patients in Radiology Ward of Mashhad's Shahid Kamyab Emergency Hospital in 2010. J Dent Mater Tech.

[26] Rafatpanah H, Hedayati-Moghaddam MR, Fathimoghadam F, Bidkhori HR, Shamsian SK, Ahmadi S (2011). High prevalence of HTLV-I infection in Mashhad, Northeast Iran: a population-based seroepidemiology survey. J Clin Virol.

[27]  Yearbook  IS (2013). Statistical Center of Iran.

[28] Arabion H, Tabrizi R, Aliabadi E, Gholami M, Zarei K (2014). A Retrospective Analysis of Maxillofacial Trauma in Shiraz, Iran: a 6-Year-Study of 768 Patients (2004-2010). J Dent.

[29] Bakardjiev A, Pechalova P (2007). Maxillofacial fractures in Southern Bulgaria–a retrospective study of 1706 cases. J Cranio Maxillo Surg.

[30] De Matos F, Arnez M, Sverzut C, Trivellato A (2010). A retrospective study of mandibular fracture in a 40-month period. Inter J Oral Maxillo Surg.

[31] Iida S, Kogo M, Sugiura T, Mima T, Matsuya T (2001). Retrospective analysis of 1502 patients with facial fractures. Inter J Oral Maxillo Surg.

[32] Joshi SR, Saluja H, Pendyala GS, Chaudhari S, Mahindra U, Kini Y (2013). Pattern and Prevalence of Maxillofacial Fractures in Rural Children of Central Maharashtra, India. A Retrospective Study. J Maxillofac Oral Surg.

[33] Kambalimath HV, Agarwal SM, Kambalimath DH, Singh M, Jain N, Michael P (2013). Maxillofacial Injuries in Children: A 10 year Retrospective Study. J Maxillofac Oral Surg.

[34] Lee JH, Cho BK, Park WJ (2010). A 4-year retrospective study of facial fractures on Jeju, Korea. J Cranio Maxillo Surg.

[35] Mijiti A, Ling W, Tuerdi M, Maimaiti A, Tuerxun J, Tao YZ (2014). Epidemiological analysis of maxillofacial fractures treated at a university hospital, Xinjiang, China: a 5-year retrospective study. J CranioMaxillo Surg.

[36] Momeni H, Shahnaseri S, Hamzeheil Z (2011). Distribution assessment of maxillofacial fractures in trauma admitted patients in Yazd hospitals: An epidemiologic study. Dent Res J (Isfahan).

[37] Arangio P, Vellone V, Torre U, Calafati V, Capriotti M, Cascone P (2014). Maxillofacial fractures in the province of Latina, Lazio, Italy: Review of 400 injuries and 83 cases. J CranioMaxillo Surg.

[38] Bali RK, Sharma P, Garg A, Dhillon G (2013). A comprehensive study on maxillofacial trauma conducted in Yamunanagar, India. J Injury Viol Res.

[39] Eidt JMS, De Conto F, De Bortoli MM, Engelmann JL, Rocha FD (2013). Associated injuries in patients with maxillofacial trauma at the hospital são vicente de paulo, passo fundo, Brazil. J Oral Maxillo Res.

[40] Maliska MC, Lima Junior SM, Gil JN (2009). Analysis of 185 maxillofacial fractures in the state of Santa Catarina, Brazil. Braz Oral Res.

[41] van Hout WM, Van Cann EM, Abbink JH, Koole R (2013). An epidemiological study of maxillofacial fractures requiring surgical treatment at a tertiary trauma centre between 2005 and 2010. Br J Oral Maxillofac Surg.

[42] Nalic B, Mijatov I, Mijatov S (2013). Epidemiology of lower jaw fracture in patients treated at the department of maxillofacial and oral surgery of the clinical centre of Vojvodina. Med Pregl.

[43] Cabalag MS, Wasiak J, Andrew NE, Tang J, Kirby JC, Morgan DJ (2014). Epidemiology and management of maxillofacial fractures in an Australian trauma centre. J PlasticRecon Aes Surg.

[44] Lee K (2012). Global trends in maxillofacial fractures. Craniomaxillo Trauma recons.

[45] Gabrielli MAC, Gabrielli MFR, Marcantonio E, Hochuli-Vieira E (2003). Fixation of mandibular fractures with 2.0-mm miniplates: review of 191 cases. J Oral Maxillo Surgy.

[46] Haug RH, Adams JM, Conforti PJ, Likavec MJ (1994). Cranial fractures associated with facial fractures: a review of mechanism, type, and severity of injury. J Oral Maxillo Surgy.

[47] Rajput D, Bariar L (2013). Study of maxillofacial trauma, its aetiology, distribution, specturm, and management. J Indian Med Assoc.

[48] Subhashraj K, Ramkumar S, Ravindran C (2008). Pattern of mandibular fractures in Chennai, India. Br J Oral Maxillofac Surg.

[49] Taher AA (1993). Management and complications of middle-and upper-third facial compound injuries: an Iranian experience. J Cranio Surg.

[50] Tanaka N, Tomitsuka K, Shionoya K, Andou H, Kimijima Y, Tashiro T (1994). Aetiology of maxillofacial fracture. Br J Oral Maxillo Surgy.

[51] Gassner R, Tuli T, Hächl O, Rudisch A, Ulmer H (2003). Cranio-maxillofacial trauma: a 10 year review of 9543 cases with 21067 injuries. J Cranio-maxillo Surg.

[52] Ugboko V, Odusanya S, Fagade O (1998). Maxillofacial fractures in a semi-urban Nigerian teaching hospital: A review of 442 cases. Inte J Oral Maxillo Surgy.

[53] Malara P, Malara B, Drugacz J (2006). Characteristics of maxillofacial injuries resulting from road traffic accidents--a 5 year review of the case records from Department of Maxillofacial Surgery in Katowice, Poland. Head Face Med.

[54] Faryabi J, Rajabi M, Alirezaee S (2014). Evaluation of the use and reasons for not using a helmet by motorcyclists admitted to the emergency ward of shahid bahonar hospital in kerman. Archives Trauma Res.

[55] Huelke DF, Compton CP (1983). Facial injuries in automobile crashes. J Oral Maxillo Surgy.

[56] Van As A, Van Loghem A, Biermans B, Douglas T, Wieselthaler N, Naidoo S (2006). Causes and distribution of facial fractures in a group of South African children and the value of computed tomography in their assessment. Inter J Oral Maxillo Surgy.

[57] Hussain K, Wijetunge DB, Grubnic S, Jackson IT (1994). A comprehensive analysis of craniofacial trauma. J Trauma Acute Care Surg.

[58] Adeyemo WL, Taiwo OA, Ladeinde AL, Ogunlewe MO, Adeyemi MO, Adepoju AA (2012). Mid-facial fractures: a 5-year retrospective review in a Nigerian teaching hospital. Niger J Med.

[59] Baylan JM, Jupiter D, Parker WL, Czerwinski M (2016). Management of Zygomatic Fractures: A National Survey. J Craniofacial Surgy.

[60] Cheema SA, Cheema SS (2016). An analysis of etiologies, patterns and treatment modalities of fracture mandible. An King Edward MedUniv.

[61] Hwang K, You SH, Kim SG, Lee SI (2006). Analysis of nasal bone fractures; a six-year study of 503 patients. J Craniofacial Surg.

[62] Renkonen S, Vehmanen S, Mäkitie A, Blomgren K (2016). Nasal bone fractures are successfully managed under local anaesthesia–experience on 483 patients. Clin Otolaryngoly.

[63] Samieirad S, Tohidi E, Pakravan M (2016). A conservative method for treating severely displaced pediatric mandibular fractures: an effective alternative technique. J Dent Mater Tech.

[64] Atisha DM, van Rensselaer Burr T, Allori AC, Puscas L, Erdmann D, Marcus JR (2016). Facial fractures in the aging population. Plastic Recon Surg.

[65] Shankar AN, Shankar VN, Hegde N, Prasad R (2012). The pattern of the maxillofacial fractures–a multicentre retrospective study. J Cranio-Maxillo Surg.

[66] Naghavi M, Shahraz S, Bhalla K, et al (2009). Adverse health outcomes of road traffic injuries in Iran after rapid motorization. Arch Iran Med.

[67]  Nikzad  F (2006). First book in road traffic injury and its damages, causes and suggestions for prevention of outcomes.

